# Community Social Pediatrics in an Underserved Canadian City: Design and Early Outcomes of an Exploratory Pilot Study

**DOI:** 10.3390/healthcare14203391

**Published:** 2026-10-12

**Authors:** Brandon d’Eon, Marianne McKenna, Natalia Fana, Sarah Lunney, Sarah Ann Campbell, Donaldo D. Canales, Deanna Merriam, Chantal MacLean, Sarah Gander

**Affiliations:** 1 Discipline of Pediatrics, Memorial University of Newfoundland, 230 Elizabeth Ave., St. John’s, NL A1B 1T5, Canadamarianne.mckenna@horizonnb.ca (M.M.); 2 Department of Pediatrics, Dalhousie University, Halifax, NS B3H 4R2, Canada; chantal.maclean@dal.ca; 3 Horizon Health Network, Saint John, NB E2L 4L2, Canada; natalia.fana@horizonnb.ca (N.F.); sarah.lunney@horizonnb.ca (S.L.); donaldo.canales@horizonnb.ca (D.D.C.);; 4 New Brunswick Social Pediatrics, 106 Princess St., Saint John, NB E2L 1K4, Canada; 5 Department of Interdisciplinary Studies, University of New Brunswick, 3 Bailey Drive, Fredericton, NB E3B 5A3, Canada; sarah.campbell@unb.ca

**Keywords:** Social Pediatrics, child health services, community health services, integrated behavioural health services, collaborative care, pediatric primary care

## Abstract

**Highlights:**

**What are the main findings?**

- Less parent/child conflict and better access to care and communication, which reflect the goals and strengths of the Community Social Pediatrics model.
- Behaviorally, there were modest improvements in externalizing behaviours, with the 1.5 year observation period possibly being too short to see measurable change.
- Barriers to “Competence” (participation in activities and social functioning) were further exacerbated by the COVID-19 pandemic, which occurred during the study period, during which community programs and extracurricular opportunities were substantially reduced or unavailable.

**What are the implications of the main findings?**

- CSP’s integrated, trauma-informed, and collaborative approach can address key limitations of traditional pediatric care pathways, particularly for children and families facing intersecting social adversities.
- The CSP approach has the potential to set children on a path towards better long-term health and improved quality of life.
- By integrating a CSP model into standard practice, Canada can move toward a proactive system that more completely supports children wherever they live.

**Abstract:**

Background: In Saint John, New Brunswick, nearly 1 in 3 children live in poverty, and the traditional pediatric care model is often ill-equipped to address the complex social realities that children experience. Community Social Pediatrics (CSP) is an interdisciplinary model that integrates health and social care, with a strong focus on children’s rights and social determinants of health. In response to increasing gaps and challenges in accessing care, a CSP-based pilot clinic was established in Saint John. The goal was to create a collaborative, child- and family-centred assessment model designed to reduce service fragmentation and improve outcomes. Methods: This paper outlines the rationale for the pilot, the design of the new clinic, and key early findings from a pre–post cohort study involving 26 children and families. Results: Significant reductions were observed in child–caregiver conflicts and behavioural challenges, while caregivers reported improved communication with healthcare providers and fewer access barriers. Conclusions: These early exploratory results suggest that CSP offers a promising care model, particularly in communities where children are at risk for being underserved by the current system. Broader adoption of CSP in Canada could meaningfully reduce health disparities, support family well-being, and lay the foundation for stronger community health outcomes over time.

## 1. Introduction

### 1.1. Pediatric Behavioural Diagnoses and the Traditional Model

Common behavioural disorders such as attention deficit hyperactivity disorder and autism spectrum disorder can negatively impact academic achievement, social interactions, and family dynamics [1]. Families increasingly face growing challenges in accessing and navigating pediatric behavioural health care services. In Saint John, New Brunswick, nearly 1 in 3 children live in poverty [2], and the traditional pediatric care model is often ill-equipped to address the complex social realities that children experience. In Saint John, New Brunswick, more than half of behaviour-related referrals to pediatric care in Saint John come from lower-income neighbourhoods, magnifying these challenges [3]. In addition to healthcare access issues, behavioural assessments become more complicated as these children are more likely to experience trauma, chronic stress, poverty, and food insecurity, which can mask or exaggerate symptoms. Children facing social disparities and adverse childhood experiences are at increased risk for negative physical and mental health outcomes across their lifespan [4,5,6,7].

Trauma-related behaviours that resemble neurodevelopmental disorders require careful consideration and are often missed by current diagnostic pathways. We have observed that children with trauma-related behaviours are often presumed to have neurodevelopmental disorders, leading to the initiation of medications, recommendations for augmented resources within school and healthcare systems, and additional services through allied health teams. Conversely, children whose symptoms do not fit a specific diagnosis frequently fail to access appropriate resources or any resources at all. The current healthcare model does not adequately identify these children, understand their circumstances, or address the underlying issues impacting their well-being [8].

Our current model also faces challenges with ensuring timely access to care and caregiver satisfaction. Referrals typically follow the traditional pathway with a primary care provider (PCP) sending children to a consultant pediatrician. Many children seen through our pediatric practice for behavioural concerns wait 12 to 18 months for an appointment, only to be redirected to other providers who are better suited to address the child’s specific needs (e.g., occupational therapists, speech-language pathologists, psychologists, etc.). This misalignment between family expectations, PCP expectations of pediatric care, and actual pediatric service provision delays effective care as families are bounced between providers without resolution. The result is a fragmented and costly system where families must navigate medical, school-based, and community support that often lack coordination and erode trust in the system and providers [8,9].

### 1.2. Community Social Pediatrics: A Nuanced Approach 

A Community Social Pediatrics (CSP) model offers an alternative approach to caring for children and families impacted by adverse childhood experiences who are seeking support for behavioural and developmental concerns. Popularized in Montreal, Canada by Dr. Gilles Julien, CSP is grounded in the 1989 United Nations Convention on the Rights of the Child [10]. The model acknowledges and responds to the complex social realities some children face, including poverty, homelessness, food insecurity, and other systemic barriers [11]. CSP emphasizes interdisciplinary collaborations where health professionals and community partners work together to address the specific needs of children and their families in the context of their communities [11]. Care delivered through a CSP model aims to reduce access barriers that are faced by families in the traditional system by reducing duplication and increasing communication and collaboration across the care team.

CSP models have demonstrated improvements in children’s academic performance, overall well-being, and individual health across the lifespan [11]. Additionally, a CSP approach may provide a more comprehensive picture of a child’s risk factors, enabling timely interventions and potentially decreasing reliance on the system over their lifespan [5].

### 1.3. The Present Study

New Brunswick Social Pediatrics (NBSP), based in Saint John, aims to improve the health, quality of life, and long-term outcomes of children and their families. NBSP provides services to children and families situated at the nexus of poverty, trauma, toxic stress, and behavioural, academic, mental health, and social issues. This study presents findings from an NBSP-led CSP pilot clinic, using an exploratory pre–post design to evaluate changes in child behavioural and emotional functioning, child–caregiver relationships, and caregiver experiences with healthcare access and communication. We explored whether the pilot clinic would be associated with meaningful improvements in each of these domains. 

## 2. Materials and Methods

This exploratory pilot study employed a prospective single-group pre–post design, following participants for approximately one year. Assessments were intended to be administered at the time of intake into the clinic and 12 months post-intake in the clinic; however, due to the COVID-19 pandemic and research staffing constraints, many of the follow-up assessments were delayed, ranging from 12 to 22 months (median 17 months). The 12 month post-intake follow-up reflects an administrative endpoint for research observation. Patients still continued to receive care following the 12 month endpoint of the research observation period. All methods and procedures described below were approved by the Horizon Health Network Human Research Protection Program/Research Ethics Board (protocol code File No: 100075). 

### 2.1. Implementing CSP in Saint John, New Brunswick

We established a pilot assessment and treatment clinic in a centrally accessible location, where nearly 50% of children live in poverty [1]. We used a “kitchen table” approach where children, their families, and key figures in their lives were invited to discuss their concerns with our team in a warm, home-like environment. The multidisciplinary team included pediatricians, social workers, primary care providers, allied health professionals, and community partners, with other professionals invited as needed based on the child’s needs. 

The goal of the kitchen table approach is to improve communication and collaboration by giving providers space to work out of the same location and conduct joint assessments. Participants received standard care alongside additional CSP-aligned resources, including ongoing social work support, home visits, and referrals for clinically indicated therapeutic services (i.e., occupational therapy) or positive childhood experiences (i.e., recreation), often with financial support to ensure families could access services that would have otherwise not been accessible. 

### 2.2. Recruitment

Participants were recruited consecutively to minimize selection bias. The first 32 participants who met eligibility criteria and received behavioural referrals made to the regional Pediatric clinic and other service providers (i.e., teachers, social workers, Occupational Therapists, etc.) were recruited to voluntarily participate in the exploratory study between November 2019 and June 2021. The eligibility criteria to participate in the study included children ranging 5–18 years of age, families who live in the Saint John and surrounding area, and at least one member of the family can speak English. 

### 2.3. Measures and Variables

To understand the experiences of children and their families receiving care through our hypothesis-generating pilot CSP clinic, caregivers completed a triage questionnaire and three measures at intake and again approximately 17 months later. Intake measures were parent-report and primarily self-administered, with research team members providing verbal administration for caregivers with low literacy. In order to comply with COVID-19 pandemic social distancing restrictions, follow-up measures were primarily verbally administered by phone. Data collection was completed in February 2023.

#### 2.3.1. Triage Questionnaire 

The questionnaire is a measure to capture the demographic information of the child; the resources the child had accessed previously, was currently accessing or was recommended; information on the mother’s pregnancy health; and the child’s development. 

#### 2.3.2. Child Behaviour Checklist 6–18 (CBCL)

The CBCL is a measure of emotional and behavioural problems in children ages 6–18. Consistent with the ASEBA guidelines, the CBCL 6–18 form can be used with children who are a few months younger or older than the suggested age range of 6–18 without causing substantial error [12]. Three of the participants included in the study were slightly younger than six years of age at intake. We used the CBCL 6–18 for all the participants to maintain consistency across the assessment. Section one collects information on demographic, family structure, child hobbies and interests, social functioning, and school performance. Section two (113 items) assesses internalizing (e.g., anxiety, depression), externalizing (e.g., aggression, rule-breaking), and other problem areas (e.g., social, attention). Items are rated on a 3-point scale (0 = not true (as far as you know); 1 = somewhat or sometimes true; 2 = very true or often true) with higher scores indicating greater difficulties. Reliability and validity are well established [13].

#### 2.3.3. The Quality of Relationship Inventory (QRI)

The QRI is a measure assessing the quality of child–caregiver relationships. It consists of 25 items across three domains: Support (perceived availability of help), Conflict (disagreements and negative interactions), and Depth (positive and secure aspects of the relationship). Items are rated on a 4-point scale (1 = not at all; 4 = very much), with higher scores indicating a more positive relationship on the Support and Depth scales, and greater strain on the Conflict scale. Reliability and validity of the QRI have been demonstrated [14].

#### 2.3.4. The Pediatric Integrated Care Survey (PICS)

The PICS is a measure assessing experiences with integrated pediatric care [15]. It includes 19 items across five domains: Access to Care, Care Planning, Family Impact, Communication (between healthcare providers and parents), and Team Functioning, with additional questions for demographic and healthcare/health status. Most items are rated on a 6-point scale (1 = never; 6 = always) with higher scores reflecting a greater perceived level of care integration. The PICS demonstrated adequate reliability and validity [16]. 

### 2.4. Data Analysis Plan

Descriptive analyses of demographics were performed for all demographics and study measures. Paired-samples *t*-tests were performed to evaluate changes for the CBCL, QRI, and PICS subscales from intake to approximately 17 months post-intake. Assumptions underlying the paired-samples *t*-tests were assessed and no major violations were identified. Cohen’s d standardized mean difference effect size (small 0.20, medium 0.50, large 0.80) was reported alongside 95% confidence intervals (CIs). Alpha for pre–post analyses was set to 0.05. This exploratory study included all eligible children enrolled in the program for whom data were available during the study period; thus, the sample size was determined by the available program population rather than a formal *a priori* sample size calculation.

## 3. Results

In total, 32 child–caregiver pairs were recruited and consented to participate in the study. Twenty-six pairs completed the follow-up assessment. Six pairs did not complete the follow-up due to withdrawal, declining further participation, or inability to be reached by the research team.

### 3.1. Demographics

Demographic data on the children and parents/caregivers were collected at intake (see Table 1). The sample included slightly more male than female children. The most common primary caregiver arrangement was a mother–father dyad, with both caregivers typically in their mid-30s. Most mothers had completed high school (or equivalent) or trade/technical education. Fathers showed greater variability in educational attainment, ranging from partial or completed high school to trades/technical training and some college.

In total, 73% (n = 19) of the participants were referred by a clinician while 26.9% (n = 7) were referred by other sources to the CSP clinic. At intake into the CSP clinic, 73% of patients were currently being seen by a family doctor, while 19.2% previously had one. A higher proportion of patients previously had a mental health provider (57.6%), while fewer were currently receiving mental health and allied health services (19.2%). Certain supports such as pediatric care, psych-eds, or occupational therapy were recommended for patients to connect with. ASD-specific services remained consistently low across previous, current, and as recommended services. Overall, families had previously accessed multiple services, but fewer had current services involved at the time of intake (Table 2).

### 3.2. CSP Model and Children

At approximately 17 months post-CSP program intake, participating children exhibited changes across a few domains. With respect to the QRI (see Table 3), significant reductions were observed in child–caregiver conflict (M_T1_ = 1.23 vs. M_T2_ = 0.87, *p* = 0.002, d = −0.689 [95% CI = −1.113 to −0.255]). 

Analysis of the responses to the CBCL (Table 4) showed significantly lower competence scores at approximately 17 months post-intake in both the activities (M_T1_ = 45.38 vs. M_T2_ = 37.92, *p* < 0.001, d = −0.923 [95% CI = −1.378 to −0.456]) and social skills (M_T1_ = 44.08 vs. M_T2_ = 40.44, *p* = 0.039, d = −0.437 [95% CI = −0.843 to −0.022]) domains. Statistically significant reductions occurred for aggressive behaviour (M_T1_ = 68.85 vs. M_T2_ = 64.77, *p* = 0.047, d = −0.410 [95% CI = −0.807 to −0.006]) and conduct problem (M_T1_ = 65.69 vs. M_T2_ = 62.31, *p* = 0.034, d = −0.440 [95% CI = −0.840 to −0.033]) subscales. No significant changes were noted among any other CBCL subscales. A number of paired comparisons were performed using an alpha of 0.05. The authors acknowledge that no correction was applied. As the analyses are exploratory, no primary outcome was prespecified, and reported *p*-values should therefore be interpreted cautiously.

### 3.3. CSP Model and Caregivers

After approximately 17 months, caregivers reported improved access to services and more effective communication with healthcare providers (Table 5). PICS scores indicated that families reported fewer access problems (M_T1_ = 3.35 vs. M_T2_ = 1.19, *p* = 0.003, d = −0.635 [95% CI = −1.052 to −0.208]) and better communication with healthcare providers (M_T1_ = 14.53 vs. M_T2_ = 16.92, *p* = 0.035, d = 0.437 [95% CI = 0.030 to 0.836]). Other PICS subscales had no significant changes.

## 4. Discussion

This exploratory study provides support for Community Social Pediatrics as an alternative holistic model for delivering pediatric care, particularly in communities that may require nuanced considerations for children facing barriers and socially complex circumstances. While improvements were observed within the relational and caregiver experience domains, changes were not uniform across all measured outcomes, highlighting both the strengths and limitations of CSP’s impact within a 1.5 year follow-up period. Regardless, our exploratory results offer important insight into how utilizing a CSP model may influence child behavioural outcomes, child–caregiver relationships, and caregiver experiences navigating the healthcare system. Taken together, these findings suggest that CSP’s integrated, trauma-informed, and collaborative approach can provide sufficient support for children and families facing intersecting social adversities.

### 4.1. Findings

Children receiving CSP care showed reductions in their aggressive behaviours and conduct problems, alongside improvements in child–caregiver relationship quality. These findings suggest that CSP may impact behavioural challenges not only through clinical intervention, but also through improved family dynamics, enhanced communication, and strengthened caregiver capacity. 

Notably, caregiver-reported scores on the CBCL competence subscales related to social skills and activities declined over the study period. Rather than reflecting a deterioration in child functioning, we hypothesize that these findings may capture structural barriers to participation that were particularly salient within this population. Anecdotally, many families served through the CSP clinic face persistent socioeconomic constraints, including limited financial resources or transportation challenges, which restrict access to organized social and recreational activities. Anecdotal reports from parents lead us to hypothesize that these barriers may have been further exacerbated during the COVID-19 pandemic, when community programs and extracurricular opportunities were substantially reduced or unavailable. 

Caregivers also reported markedly fewer access barriers and improved communication with healthcare providers. These caregiver observations may speak to the described benefits of a CSP model: its ability to deliver coordinated, timely, and relationally grounded care. Importantly, the domains demonstrating the greatest change, child aggressive behaviour, conduct problems, caregiver–child conflict, and caregiver-reported healthcare access and communication, align closely with the relational and structural targets of CSP. The findings suggest that this model may improve emotional stability, functioning relationships, and health system navigation rather than symptom resolution alone [11].

### 4.2. Alignment with Current Literature

The findings of this exploratory study align with and extend the existing literature on integrated and collaborative care models such as CSP, which emphasize the importance of addressing behavioural concerns within the broader social and relational contexts of children’s lives [17,18,19,20]. We reinforce that integrated and socially responsive pediatric care models may improve both relationship quality and behavioural outcomes. Prior research has demonstrated that applying a pediatric collaborative care approach was associated with a decrease in parent-reported conflicts between parents and their children, and less parental distress related to their child’s behaviour [17]. Similarly, a 2020 systematic review of pediatric collaborative care models found that collaborative care approaches that improved access to care improved mental health and behavioural outcomes while also increasing caregiver and provider satisfaction, particularly in populations experiencing complex social needs [18]. 

Qualitative evidence emerging from the Atlantic Canadian context further strengthens the interpretation of our results. Plourde et al. (2025) [21] explored caregiver perceptions of CSP services delivered in rural New Brunswick, finding that caregivers valued the model’s accessibility, flexibility, and collaborative nature, reporting perceived improvements in their children’s behavioural functioning as well as in family and peer relationships [21]. Caregivers emphasized the importance of trust, continuity, and coordinated service delivery, which were closely mirrored by the reductions in caregiver–child conflict and improvements in healthcare access and communication observed in this study.

Importantly, Plourde et al. (2025) [21] also situate CSP within the broader social realities facing families in New Brunswick, including elevated rates of poverty, mental health challenges, and limited access to services, particularly in rural and underserved communities. Within this context, perceived improvements in children’s emotional regulation and relational functioning were understood as early forms of skill development that support coping in the face of adversity. Our findings complement these observations by quantitatively demonstrating that implementing a CSP model may be associated with measurable improvements in relational and behavioural domains, even within a relatively short follow-up period. 

Improvements in caregiver-reported access and communication further align with CSP’s core principles and with the recent Canadian Pediatric Society recommendations emphasizing supportive, collaborative, and family-centred mental healthcare [11,19]. Together, our findings suggest that CSP represents a structurally innovative model of pediatric care for children facing intersecting social adversities.

### 4.3. Implementing a CSP Model in Local Contexts 

The behavioural, relationship, and healthcare access improvements observed in this exploratory study illustrate possible mechanisms through which a CSP model functions as a holistic model for pediatric care in socially complex or unique contexts. Our hypothesis-generating pilot clinic in Saint John demonstrated that CSP principles can be applied outside of large urban Canadian centres like Vancouver, Ottawa, and Montreal. Our findings complement a previous New Brunswick study showing that a CSP model can be implemented in rural areas with reasonable fidelity, provided there are adaptable processes and community engagement [20].

The identification of low social skill and activity gaps from CBCL scores may highlight a core strength of the CSP model: its capacity to adapt service delivery in response to identified needs. As the social and activity-related challenges were revealed through assessment and ongoing family engagement, our clinic responded by designing and facilitating non-traditional interventions such as a youth cooking program (“Cooking Up Justice”) and a social–emotional skills group for ages 8–12 (“Creative Expressions”) [22]. Additionally, we expanded our programming to facilitate no-cost access to therapeutic and recreational support through the “Brighter Horizons” program, a support fund designed to provide positive childhood experiences to marginalized children and youth in our community. These programs were developed in direct response to family-identified needs and are now integrated into care planning, illustrating CSP’s flexibility and commitment to addressing both clinical and social dimensions of child well-being.

Traditional referral-based pediatric care does not routinely offer these advantages, which can exacerbate delays, misalignment, and caregiver frustration [8,11]. These findings have important implications for pediatric care in local contexts such as within New Brunswick. The high prevalence of ACEs, poverty, and trauma in Saint John requires a model that can integrate medical and social supports seamlessly. Adopting CSP more broadly may reduce long-term system costs by ensuring timely intervention and reducing the need for repeated assessments, emergency care, or misaligned referrals. Ultimately, our pilot’s improvements in both child outcomes and caregiver experience suggests that CSP may address these structural shortcomings effectively.

### 4.4. Limitations and Future Research

This exploratory study has several limitations that should be considered when interpreting the findings. Follow-up assessments were delayed beyond the intended 12-month period due to the COVID-19 pandemic and research staffing constraints, which may have influenced outcome trajectories. Pandemic-related restrictions also limited the availability of social and recreational activities within the community, potentially affecting caregiver-reported outcomes related to children’s social engagement and participation in activities. Additionally, the modest sample size limits the generalizability of the findings. In the absence of a concurrent control group, changes observed over time cannot be attributed to the intervention with certainty. As a result, changes could potentially be attributed to maturation, regression to the mean, COVID-19 related effects, and other temporal influences. 

Future research should examine whether the integration of structured social, recreational, and skills-based programming within CSP clinics addresses the observed competence-related outcomes, such as children’s social skills and participation in activities over longer follow-up periods. Longitudinal studies would be particularly valuable in assessing whether embedding these programs directly into CSP care models leads to sustained improvements in social functioning, relationships, and overall well-being, especially among children facing socioeconomic barriers to accessing extracurricular opportunities. Additionally, comparative and cost-effectiveness analyses are needed to evaluate CSP relative to traditional pediatric care pathways. Such evaluations should also consider the complexity of care and the professional resources required for each child, using standardized pediatric care data where possible, as these dimensions may provide a more complete account of resource use and its relationship with outcomes [23]. Future studies could assess whether CSP reduces system-level inefficiencies, including fragmentation, service duplication, or prolonged wait times. Evidence demonstrating improved efficiency and cost savings would strengthen the case for broader policy adoption and sustained funding, while also confirming CSP’s potential to deliver both clinically effective and economically responsible pediatric care.

## 5. Conclusions

The Saint John CSP pilot demonstrates that a rights-based, interdisciplinary, and socially informed model of care can address long-standing inequities in pediatric health. By redefining care as collaborative and community-embedded, CSP may strengthen relationships, improve behavioural outcomes, and remove systemic barriers preventing children from accessing meaningful care. However, in the absence of a concurrent control group, changes observed over time cannot be attributed to the intervention with certainty. Yet, this approach has the potential to set children on a path towards better long-term health and improved quality of life. By integrating a CSP model into standard practice, Canada can move toward a proactive system that more completely supports children wherever they live.

## Figures and Tables

**Table 1:** Demographics (n = 26).

	M	(SD)	n	(%)
Child				
Age, current	8.80	(2.63)	26	(100)
Sex, Male			14	(53.8)
Sex, Female			12	(46.2)
Primary caregiver				
Mother and father			19	(73.1)
Mother or father and other			7	(26.9)
Mother				
Age, current	34.95	(6.41)	23	(88.4)
Age at pregnancy	25.15	(6.71)	26	(100)
Mother’s highest education				
Less than high school			0	(0.0)
Some high school			5	(19.2)
High school graduate or equivalent			8	(30.1)
Trade/technical			8	(30.1)
Some college			3	(11.5)
Bachelor’s degree			1	(3.8)
Father				
Age, current	35.66	(9.92)	24	(92.3)
Father’s highest education				
Less than high school			1	(4.0)
Some high school			6	(24.0)
High school graduate or equivalent			6	(24.0)
Trade/technical			4	(16.0)
Some college			7	(26.9)
Bachelor’s degree			1	(4.0)

Note: Only 21 fathers were identified as the primary caregiver. However, 24 responses were recorded for the father’s age, and 25 were recorded for fathers highest level of education.

**Table 2:** Previous, current, and recommended services at intake (n = 26).

	Previous	Current	Recommended
Services	%	%	%
Family doctor	19.2	73.0	-
Pediatrician	30.7	11.5	15.3
Mental Health	57.6	19.2	7.6
Psych-ed assessment	3.8	-	3.8
Early intervention	23.0	3.8	-
Speech language pathologist	38.4	11.5	-
Occupational therapy	19.2	-	7.6
Physiotherapy	19.2	-	-
ASD services	7.6	-	-
Other services	26.9	11.5	7.6

Note: Responses are not mutually exclusive. ASD = Autism Spectrum Disorder services. Other services included pediatric neurology, Family and Child Education (FACE), Social Development, etc.

**Table 3:** Pre–post changes in relationship quality (n = 26).

	Program Intake		~17-Month Follow-Up				
QRI	M	SD	M	SD	*p*	d	95% CI
Support	1.63	0.71	1.79	0.67	0.295	0.210	−0.181, 0.596
Conflict	1.23	0.71	0.87	0.52	0.002	−0.689	−1.113, −0.255
Depth	2.72	0.26	2.56	0.30	0.0504	−0.412	−0.817, 0.001

Note: QRI = Quality in Relationships Inventory. Depth subscale based on n = 25 due to missing data.

**Table 4:** Pre–post changes in child behaviour.

CBCL	Program Intake		~17-Month Follow-Up				
	M	SD	M	SD	*p*	d	95% CI
Competence							
Activities	45.38	7.96	37.92	6.86	<0.001	−0.923	−1.378, −0.456
Social	44.08	9.41	40.44	6.46	0.039	−0.437	−0.843, -0.022
School	36.33	7.74	36.96	7.40	0.691	0.082	−0.320, 0.482
Syndromes							
Anxious/Depressed	64.96	10.87	63.85	9.85	0.598	−0.105	−0.489, 0.282
Withdrawn	60.92	9.63	60.15	7.76	0.662	−0.087	−0.471, 0.299
Somatic	59.50	8.23	57.65	7.45	0.241	−0.236	−0.623, 0.156
Social	64.08	8.77	62.23	7.47	0.275	−0.219	−0.606, 0.172
Thought	65.77	9.70	67.23	7.70	0.413	0.163	−0.225, 0.549
Attention	65.27	10.44	64.35	8.90	0.687	−0.080	−0.464, 0.306
Rule Breaking	63.04	11.59	60.81	7.75	0.113	−0.322	−0.714, 0.075
Aggressive	68.85	14.27	64.77	10.88	0.047	−0.410	−0.807, −0.006
Internalizing	62.96	10.51	61.69	9.59	0.516	−0.129	−0.514, 0.258
Externalizing	65.27	12.88	63.00	10.62	0.142	−0.297	−0.687, 0.099
Total Problems	66.69	9.60	65.69	7.73	0.496	−0.136	−0.520, 0.252
DSM-Oriented							
Depressive	63.85	9.47	63.31	7.25	0.767	−0.059	−0.443, 0.327
Anxiety	65.15	11.20	64.50	9.31	0.758	−0.061	−0.445, 0.324
Somatic	58.00	8.85	56.19	7.73	0.264	−0.224	−0.611, 0.167
ADHD	65.19	10.94	64.27	8.94	0.649	−0.090	−0.475, 0.296
ODD	65.77	9.46	63.92	9.52	0.165	−0.281	−0.670, 0.114
Conduct	65.69	12.78	62.31	8.46	0.034	−0.440	−0.840, −0.033
2007 Scales							
Sluggish	60.00	8.13	59.69	8.30	0.889	−0.028	−0.412, 0.357
OC Problems	68.58	11.24	66.27	9.76	0.291	−0.212	−0.598, 0.179
Stress	69.35	10.84	67.73	8.88	0.376	−0.177	−0.563, 0.212

Note: CBCL = Child Behaviour Checklist; DSM = Diagnostic and Statistical Manual of Mental Disorders; ADHD = attention deficit hyperactivity disorder; ODD = oppositional defiant disorder; OC = obsessive compulsive. Competence Social and School subscales based on n = 25 and n = 24, respectively, due to missing data.

**Table 5:** Pre–post changes in integrated care perceptions (n = 26).

PICS	Program Intake		~17-Month Follow-Up				
	M	SD	M	SD	*p*	d	95% CI
Access to Care	3.35	3.10	1.19	2.33	0.003	−0.635	−1.052, −0.208
Care Goals	0.76	0.78	1.16	0.85	0.116	0.327	−0.079, 0.726
Family Impact	6.00	5.89	9.85	6.82	0.054	0.397	−0.006, 0.793
HCP Comm.	14.54	5.69	16.92	3.26	0.035	0.437	0.030, 0.836
Team function	15.65	7.63	18.08	6.33	0.191	0.264	−0.130, 0.652

Note: PICS = Pediatric Integrated Care Survey; HCP Comm. = health care provider communication. Care Goals subscale based on n = 25 due to missing data. Five factor categories: Access to Care; Care Goal Creation/Planning; Family impact; Communication between HCP (Health Care Provider) and Parent, and Team Functioning/Performance/Quality/Connectivity.

## Data Availability

The datasets generated and/or analyzed for the current project are not available publicly due to privacy and confidentiality concerns, but are available from the corresponding author on reasonable request.

## Abbreviations

The following abbreviations are used in this manuscript:

CSP	Community Social Pediatrics
PICS	Pediatric Integrated Care Survey
CBCL	Child Behavioural Check List
